# An Emerging Role of Extracellular Traps in Chronic Rhinosinusitis

**DOI:** 10.1007/s11882-023-01082-1

**Published:** 2023-11-07

**Authors:** Siyuan Zhang, Zhenlin Wang

**Affiliations:** Department of Otorhinolaryngology Head and Neck Surgery, Xuanwu Hospital, Capital Medical University, Beijing, China

**Keywords:** Chronic rhinosinusitis, Extracellular traps, Neutrophil extracellular traps, Eosinophil extracellular traps

## Abstract

**Purpose of Review:**

Chronic rhinosinusitis (CRS) is a complicated, heterogeneous disease likely caused by inflammatory and infectious factors. There is clear evidence that innate immune cells, including neutrophils and eosinophils, play a significant role in CRS. Multiple immune cells, including neutrophils and eosinophils, have been shown to release chromatin and granular proteins into the extracellular space in response to triggering extracellular traps (ETs). The formation of ETs remains controversial due to their critical function during pathogen clearance while being associated with harmful inflammatory illnesses. This article summarizes recent research on neutrophil extracellular traps (NETs) and eosinophil extracellular traps (EETs) and their possible significance in the pathophysiology of CRS.

**Recent Findings:**

A novel type of programmed cell death called ETosis, which releases ETs, has been proposed by recent study. Significantly more NETs are presented in nasal polyps, and its granule proteins LL‐37 induce NETs production in CRS with nasal polyps (CRSwNP) patients. Similar to NETs, developed in the tissue of nasal polyps, primarily in subepithelial regions with epithelial barrier defects, and are associated with linked to elevated tissue levels of IL-5 and *S. aureus* colonization.

**Summary:**

This article provides a comprehensive overview of NETs and EETs, as well as an in-depth understanding of the functions of these ETs in CRS.

## Introduction

The discovery of neutrophil extracellular traps (NETs), which are released following neutrophil cell activation, was publicly disclosed in 2004 [1]. NETs contribute to the clearance of pathogens via cytotoxic histones and granular proteins that are independent of phagocytosis. Later, the same group described a new neutrophil programmed cell death (i.e., NETosis) that releases NETs [2]. It has been observed that neutrophils, eosinophils, mast cells, and monocytes employ the production of extracellular traps (ETs) as an effective host defense mechanism. Diverse forms of ETs have been characterized as capable of binding and eliminate pathogens, including bacteria, parasites, and fungi. In addition, ETs could contribute to immunopathology in chronic inflammatory diseases such chronic obstructive pulmonary disease (COPD), chronic rhinosinusitis (CRS) and asthma [3].

CRS is a chronic inflammation of the sinonasal mucosa that significantly negatively affects the quality of life and daily functioning of the patient. It is clinically distinguished between CRS with (CRSwNP) and without (CRSsNP) nasal polyps. CRSwNP is generally the more severe phenotype and characterized by type 2 inflammation (eosinophilic predominant), while CRSsNP is usually classified as type 1/3 neutrophilic inflammation (neutrophilic predominant) [4••]. As knowledge of the pathological mechanisms of CRS advances, CRS is currently divided into two subtypes: eosinophilic chronic rhinosinusitis (ECRS) and non-eosinophilic chronic rhinosinusitis (non-ECRS). Compared to non-ECRS, ECRS presents with more severe symptoms and has a higher recurrence rate after treatment. Eosinophils are capable of secreting cytotoxic granule proteins that cause tissue damage and remodeling [5]. Eosinophils secrete their granule proteins by piecemeal degranulation, cytolysis, classical exocytosis, and compound exocytosis [6]. Recent research has revealed that activated eosinophils can also display extracellular trap cell death and produce eosinophil extracellular traps(EETs) [7]. EETs are composed of DNA, granule proteins, and are accompanied by Charcot-Leyden crystals (CLCs) [8]. The presence of EETs has been observed in subepithelial regions of nasal polyps, and is correlated with persistent eosinophilic inflammation [9]. Neutrophils play a prominent role in non-type 2 CRSwNP and CRSsNP, but a recent study has shown that neutrophils also contribute to the pathogenesis of type 2 CRSwNP [10]. As innate effector cells, neutrophils function mainly through phagocytosis, degranulation, and NETs. NETs are substantially more prevalent in nasal polyps than healthy control and correlate with neutrophil infiltration in CRSwNP patients [11]. In contrast, a different study found that none of the CRS patients containing neutrophils formed NETs in their study [12]. Consequently, the involvement of NETs in chronic rhinosinusitis remains controversial.

This article reviews current findings on ETs, particularly NETs and EETs. This study also aims to outline our present understanding of the pathophysiologic mechanisms of CRS, with an emphasis on the involvement of NETs and EETs (Table 1).

**Table 1:** Overview of NETs or EETs formation in patients with CRS

**Year**	**Authors**	**Type**	**Main study findings**
2016	Ueki et al. [88]	EETs	Eosinophilic secretions are abundant with EETosis-derived DNA traps, which contributes to an enhanced viscosity.
2017	Gevaert et al. [9]	EETs	EETs predominantly form in subepithelial locations of nasal polyp tissue, particularly where epithelial barrier defects exist, and are associated with elevated IL-5 tissue concentrations and *S. aureus* colonization.
2019	Cao et al. [11]	NETs	NETs are markedly elevated in nasal polyps, and LL-37 provokes NETs formation in patients with CRSwNP.
2019	Hwang et al. [12]	EETs	EETs formation is strongly correlated with disease severity in chronic rhinosinusitis, irrespective of the presence of NP.
2020	Wan et al. [51•]	NETs	NETs formation is augmented in exacerbated CRS, which induces chemokine secretion, enhances the epithelial barrier, and promotes neutrophil infiltration.
2020	Delemarre et al. [115]	EETs	EETs and CLCs deposition are also evident in CRSsNP and are strongly associated with the underlying type 2 inflammation.
2020	Delemarre et al. [57]	NETs	Neutrophils demonstrate a reduced propensity to generate NETs in CRSwNP tissue, and NETosis in CRSwNP is correlated with bacterial colonization.

## Neutrophil Extracellular Traps

Neutrophils are the predominant innate immune cells in the human immune system and perform an essential role in protecting the body against infection. A range of antibacterial compounds are stored in specific protein granules on these cells. In response to a wide variety of foreign antigens, including bacteria, viruses, and fungi, they deploy multiple host defensive systems. This includes phagocytosis, degranulation, the formation of reactive oxygen species (ROS) and NETosis. Neutrophils are able to efficiently respond to a wide range of infections due to their complicated and varied activities [13].

In 1996, Takei et al. were the first to report the discovery of a novel form of neutrophil cell death triggered by stimulation with phorbol 12-myristate 13-acetate (PMA). It was initially believed to be a distinct type of programmed cell death, separate from apoptosis and necrosis [14]. In 2004, NETs were identified as extracellular structures released by activated neutrophils, consisting of granule proteins and chromatin with diameters up to 50 nm [1]. Extracellular DNA (eDNA) fibers and its associated histones make up the structure of NETs, which are linked by variable granular proteins that depend on the stimulus, including neutrophil elastase (NE), cathepsin G, defensins, NADPH oxidase, myeloperoxidase (MPO), and the antimicrobial protein LL-37 [15, 16]. The formation of NETs is a heterogeneous process that can be triggered by a range of biological and synthetic stimuli, such as PMA, lipopolysaccharides (LPS), bacteria, cigarette smoke, and environmental factors. A study by Petretto et al. used proteomic analysis to examine the protein composition of NETs induced by PMA, calcium ionophore A23187, *Escherichia coli*, LPS, or without stimulation. They found that while there is a common core of components present in NETs, the different stimuli result in differential expression of proteins. The composition of PMA- and A23187-induced NETs was similar, while that of LPS-induced and spontaneous NETs showed significant differences. Furthermore, the post-translational modifications of the proteins involved in NETs formation varied with the stimulus, with methionine sulfoxidation being the most common modification, especially in PMA- and LPS-induced NETs, and MPO being the most extensively modified protein. This suggests that different stimuli can influence the protein composition and post-translational modifications involved in NETs formation, leading to the possibility of distinct biological activities in NETs induced by different stimuli [17–19].

**Fig. 1:**
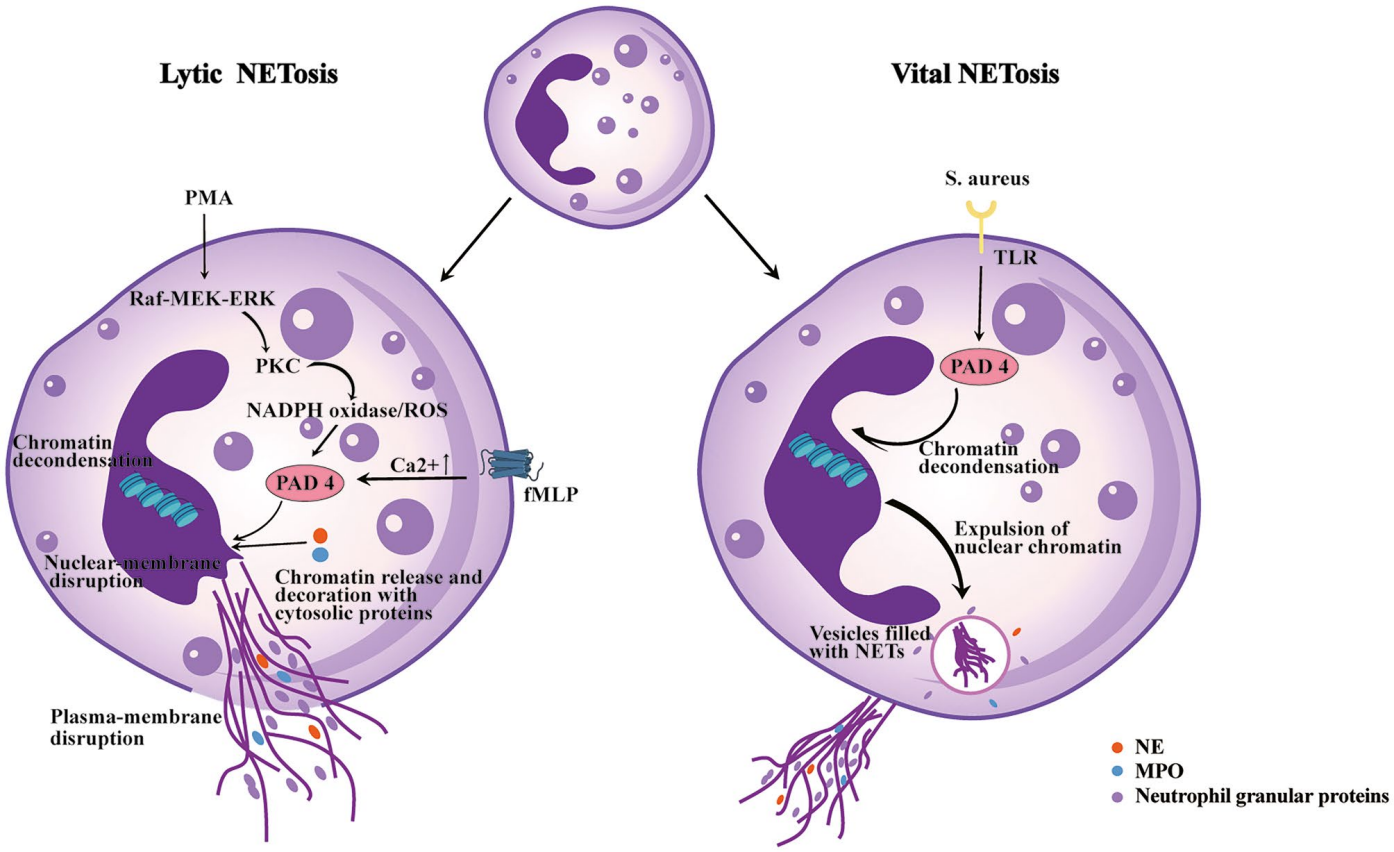
Overview of NETs formation mechanisms in neutrophils: (Left) Various stimuli, such as phorbol myristate acetate (PMA), induce suicidal NETosis, which occurs after hours of stimulation. Reactive oxygen species (ROS) are generated, and peptidylarginine deiminase 4 (PAD4) is activated following the activation of NADPH oxidase, leading to chromatin decondensation. Neutrophil elastase (NE) and myeloperoxidase (MPO) are then translocated into the nucleus to promote further chromatin unfolding, resulting in the disintegration of the nuclear membrane. Chromatin is released into the cytoplasm, where it is decorated with granular and cytosolic proteins. Ultimately, NETs are expelled through plasma membrane rupture, and the neutrophil perishes. (Right) Staphylococcus aureus elicits vital NETosis through Toll-like receptor 2 (TLR2) ligands within minutes. PAD4 is activated, potentially without oxidants, and induces chromatin decondensation. The inner and outer nuclear membranes separate and expel nuclear chromatin. Finally, protein-coated chromatin is extruded via vesicles, and the neutrophil survives to perform additional functions

Neutrophils can release NETs structures via lytic NETosis and vital NETosis processes, which depend on the presence or absence of ROS produced by NADPH oxidase [20, 21] (Fig. 1). The predominant mode of NET release is lytic or suicidal NETosis, which lasts for 2–4 h and is initiated by activation of complement and Toll-like receptors (TLR) [1, 22–24]. When neutrophil surface receptors are stimulated, the Raf-MEK-ERK pathway and PKC are activated, resulting in the activation of NADPH oxidase and ROS production [25]. This is a main mechanism of NADPH oxidase activation during NETosis. In addition, Vorobjeva et al. identified an additional signaling pathway in which an increase in mitochondrial ROS (mtROS) and activation of NADPH oxidase was triggered by a signal from the G-protein-coupled fMLP receptor, which triggered the release of Ca^2+^ from intracellular reticulum and Ca^2+^-independent activation of PI3K. The mtROS also increased NADPH oxidase with the help of PKC, but their primary target remained uncertain [26]. Next, ROS cause azurophilic granules to rupture and produce NE and MPO [27]. Granules and the nuclear envelope break under the action of ROS, and peptidyl arginine deiminase 4 (PAD4) are activated [28]. NE and MPO translocate to the nucleus and break histones while PAD4 citrullinates histones, resulting in chromatin decondensation [29]. PAD4, NE, and MPO are all necessary for the creation of NETs, which can be decreased by inhibiting any of these three components [27, 29, 30]. In the last phase, the plasma membrane ruptures and NETs are released, resulting in cell death [2].

A novel mechanism for the genesis of NETs has been identified by Clark et al. in 2007, which has been termed “vital NETosis” [31]. Unlike lytic NETosis, vital NETosis is characterized by the release of NETs without the loss of nuclear or plasma membrane integrity and without the involvement of ROS or the c-Raf/MEK/ERK pathway. Neutrophils remain viable during this process within 5–60 min, which is triggered by stimuli recognition through TLR and the complement receptor for the C3 protein [31–33]. PAD4 is activated, perhaps without any need for ROS, and induces chromatin decondensation [34]. Briefly, this process involves the condensation of the multi-lobed nucleus and separation of the inner and outer nuclear membranes, leading to the extrusion of nuclear DNA-filled vesicles into the extracellular space before their rupture and release of chromatin. In addition, there is evidence that neutrophils undergoing vital NETosis retain chemotaxis and phagocytic function [33].

NETs have been demonstrated to have potent antimicrobial effects against various microorganisms. They contain components with bactericidal properties, such as histones, cathepsin G, MPO, lactoferrin, LL-37, pentraxin 3, and peptidoglycan-binding proteins, and have been shown to limit the growth or kill bacteria, including *Pseudomonas aeruginosa*, *S. aureus*, *Propionibacterium*, and others [35]. Neutrophil elastase is recognized as a potent antimicrobial factor, which exerts its effects through direct penetration and disruption of bacterial membranes via its cationic charge [36]. Additionally, LL-37 has been shown to exhibit antimicrobial activity against a wide range of bacterial species [37]. In viral infections, such as influenza, HIV, and respiratory syncytial virus, excessive neutrophil recruitment occurs, and these viruses stimulate NETosis through TLR 4, 7, or 8, resulting in the release of ROS species and trapping, containment, and elimination of the viruses [38–40]. Additionally, histones play a role in aggregating and neutralizing viruses, leading to a decrease in viral replication [41, 42]. Fungi, such as *Aspergillus fumigatus*, *Candida albicans*, induce NETosis through recognition of β-glucan on hyphae or activation of NADPH oxidase [43–45]. Although most studies on NETs have been done in mice and in vitro, there is still limited knowledge of their exact antimicrobial mechanism in vivo, which calls for more research to evaluate their effects in vivo and in humans.

NETs are associated with various pathological conditions. While NETs help clear pathogens, they can also cause harm through the release of eDNA and proteins, leading to an uncontrolled inflammatory response and tissue damage [17, 46]. NETs have been found to exacerbate cancerous conditions by capturing metastatic tumors, as well as hinder wound healing in cases of diabetes [47, 48]. The interaction between neutrophils and platelets, mediated by P-selectin, results in the production of platelet-derived high-mobility group protein B1 (HMGB1), which promotes NETs, causing occlusion in the vasculature and organ damage [31, 49]. Surprisingly, an accumulation of NETs aggregates has been shown to reduce inflammation in a mouse model of gout by degrading cytokines and chemokines [50]. Additionally, it has been discovered that NETs induce airway cells to express CXCL1, CXCL2, and CXCL8 through the TLR 4/ NF-κB pathway, thus recruiting neutrophils to the site of inflammation. Furthermore, suppression of NETs formation reduced recruitment of lung neutrophils and neutrophilic inflammation [51•]. In conclusion, there is still much to learn about NETs, and supplemental research is required to fully comprehend their mechanisms and discover ways to take advantage of their benefits while minimizing their adverse effects.

A balance between NET production and clearance is essential for tissue homeostasis. NETs remain active for several days during infection and are typically degraded by the plasma nuclease DNase I [52]. Although injecting DNase I during *S. aureus* infection can lead to rapid elimination of NETs-associated DNA, the mechanisms for endogenous clearance of NETs are still not fully understood [53]. DNase I facilitates the ingestion of NETs by macrophages in vitro; thus, these mechanisms may involve macrophage scavenging [54]. The cleavage of chromatin into smaller fragments by DNase I enables macrophages to consume NETs remnants. Interaction with NETs causes M2 macrophages to release chemotactic mediators, activating M1 macrophages and monocytes, which in turn promotes the breakdown of NETs. With the aid of LL-37, M2 macrophages also efficiently engulf and digest fragments through an active endocytosis process [54–56]. Further studies are required to elucidate the mechanisms for treatment related NETs.

## Neutrophil Extracellular Traps in CRS

Neutrophils and NETs are vital to the pathogenesis of CRS. Neutrophils are normally prevalent in 50% of CRSsNP, and they have also been implicated in severe type 2 CRSwNP disease. In CRSwNP patients, mature neutrophils are prevalent in the blood, but a significant shift of activated neutrophils is observed in the tissue, indicating that they get activated when they enter the CRSwNP environment [57, 58]. Activated neutrophils help fight bacterial infections through phagocytosis of *S. aureus* and oxidative burst, and are involved in the development of airway hyperreactivity [59, 60]. Several studies have shown elevated levels of proteolytic activity from both NE and cathepsin G granule proteins secreted by activated neutrophils in the tissue of type 2 CRSwNP patients [57, 58]. These proteins are less effective at killing microorganisms, but they can increase the secretion and activation of IL-1 family cytokines such as IL-1, IL-33, and IL-36 [61]. The degradation of elastin, collagen, and fibronectin, which are major components of the extracellular matrix, is linked to tissue remodeling and is caused by neutrophil proteases such as NE [62]. Additionally, neutrophil serine proteases can have a direct harmful effect on the integrity of the nasal epithelial barrier and can cause goblet cell metaplasia and increased mucus production [63, 64]. NETs, made up of neutrophil DNA and granule proteins, are abundant in the subepithelial regions of CRSsNP and CRSwNP patients’ tissues and secretions [11, 65]. Recent research has shown that NETosis is primarily found at the edges of the epithelium and is colocalized with signs of bacterial colonization in CRSwNP. In CRSsNP tissue, NETosis is mainly found in the stroma and underneath a clear, thickened basement membrane associated with depleted epithelium. The release of NETs is highly influenced by the type of microbe, the size of the pathogen, and various other stimuli [35, 66]. For example, LL-37 was shown to stimulate NETs formation in CRSwNP patients [11]. Another study showed that CLCs can induce NETosis in vitro, suggesting that CLCs in CRS patients’ tissues and secretions may contribute to tissue damage [66, 67]. Moreover, *S. aureus* was found in the majority of CRSwNP cases and was seen to degrade NETs, promoting its own survival [68]. Wang et al. discovered a novel antibacterial mechanism in which bacterial infection causes the production of the alarm cytokine IL-33 in lesion tissues, activating neutrophils to form NETs and enhancing the host’s innate defense against the infection. Furthermore, the formation of NETs by IL-33-primed neutrophils after bacterial exposure depends on classical ROS generation from NADPH oxidase [69]. On the other hand, Hwang et al. found that none of the CRS groups containing neutrophils developed NETs in the subepithelial or stroma regions [12]. Consequently, the presence of NETs in CRS tissues still have controversial.

It is well established that the overproduction of NETs and their ineffective clearance can result in tissue damage and inflammation. Multiple studies have demonstrated that NETs contribute to the worsening of airway inflammation and epithelial damage. In particular, NETs present in the secretions of patients with eosinophilic CRSwNP have been shown to increase the viscosity of mucus, causing plug formation, hindering mucociliary clearance, and ultimately leading to airway damage [70]. NETs have been found to trigger hypersecretion of mucus in airways in animal studies [71]. Saffarzadeh et al. discovered that NETs can directly cause the death of human epithelial and endothelial cells, suggesting that they have toxic properties. The cytotoxic properties of NETs are thought to be associated with their components, with histones being particularly responsible for the cytotoxic effect [72]. In severe asthma patients, elevated eDNA levels in sputum have been associated with increased CXCL-8, IL-1β, and caspase-1 activity. This association may be due to the ability of NETs to activate the inflammasome in cells such as monocytes and macrophages, which triggers the secretion of IL-1β. The release of IL-1β promotes neutrophil recruitment to the lung, further perpetuating the cycle of inflammation [73–75]. Neutrophil recruitment by NETs is also amplified by the stimulation of airway epithelial cells to express CXCL-1, CXCL-2, and CXCL-8 via the Toll-like receptor 4/NF-κB pathway [51•]. Furthermore, recent evidence suggests that in a subset of patients with severe asthma, NETs-mediated inflammation by neutrophil cytoplasts may drive immune responses toward Th17-associated inflammation [76, 77]. In addition, it has been proposed that NETs may also contribute to the development of a type 2 immune response. Studies have shown that rhinovirus, which is commonly found in patients with chronic rhinosinusitis, can stimulate the release of double-stranded DNA (dsDNA) connected with NETs formation. This process can be inhibited by blocking NE or by using DNase treatment to degrade the NETs. Additionally, research in mice has demonstrated that administration of endogenous dsDNA can lead to a type 2 immune response mediated by T-helper cells, indicating that NETs formation plays a direct role in type 2-mediated inflammation [78, 79]. In conclusion, the evidence suggests that NETs play a significant role in the severity of airway epithelial damage and inflammation in the pathogenesis of CRS. However, the role of NETs in the pathophysiology and persistence of CRS, particularly in a type 2 context, remains largely unclear and requires further investigation to improve treatment and patient endotype.

## Eosinophil Extracellular Traps

Eosinophils are known for their role in host responses to helminth infections, and as effector cells in allergic illnesses such as atopic dermatitis, asthma, eczema, and allergic rhinitis. Additionally, they have been linked to non-allergic diseases, including Crohn’s disease, COPD, and non-atopic asthma. Eosinophils produce potent immunomodulatory substances stored within their granules [80]. These granules contain preformed stores of major granule proteins, such as major basic protein(MBP), eosinophil cationic protein(ECP), eosinophil-derived neurotoxin(EDN), and eosinophil peroxidase(EPX), which are cationic proteins that are harmful to both external pathogens and host tissue [70]. Eosinophils release these granules through classical exocytosis, compound exocytosis, and piecemeal degranulation. It is believed that the secretion of granular components by tissue-dwelling eosinophils is a key mechanism in eosinophilic inflammatory diseases.

The concept of eosinophils releasing ETs was first introduced in 2008, 4 years after the discovery of extracellular trap release by neutrophils. Yousefi et al. conducted a study that revealed the existence of several eDNA fibers linked to ECP and MBP in colon biopsies obtained from patients with schistosomiasis, Crohn’s disease, or intestinal spirochetosis. In vitro experiments showed that human eosinophils primed with IL-5 or IFN-γ and activated with C5a, LPS, or eotaxin produced ETs via a ROS-dependent mechanism. The DNA found in the ETs was identified as originating from mitochondria, and the process did not involve cell death [7]. EETs consist of chromatin fibers with a diameter of 25–35 nm, which are encapsulated by histone. The release of granular proteins, cell-free intact granules, and the formation of CLCs also accompany the formation of EETs.

**Fig. 2:**
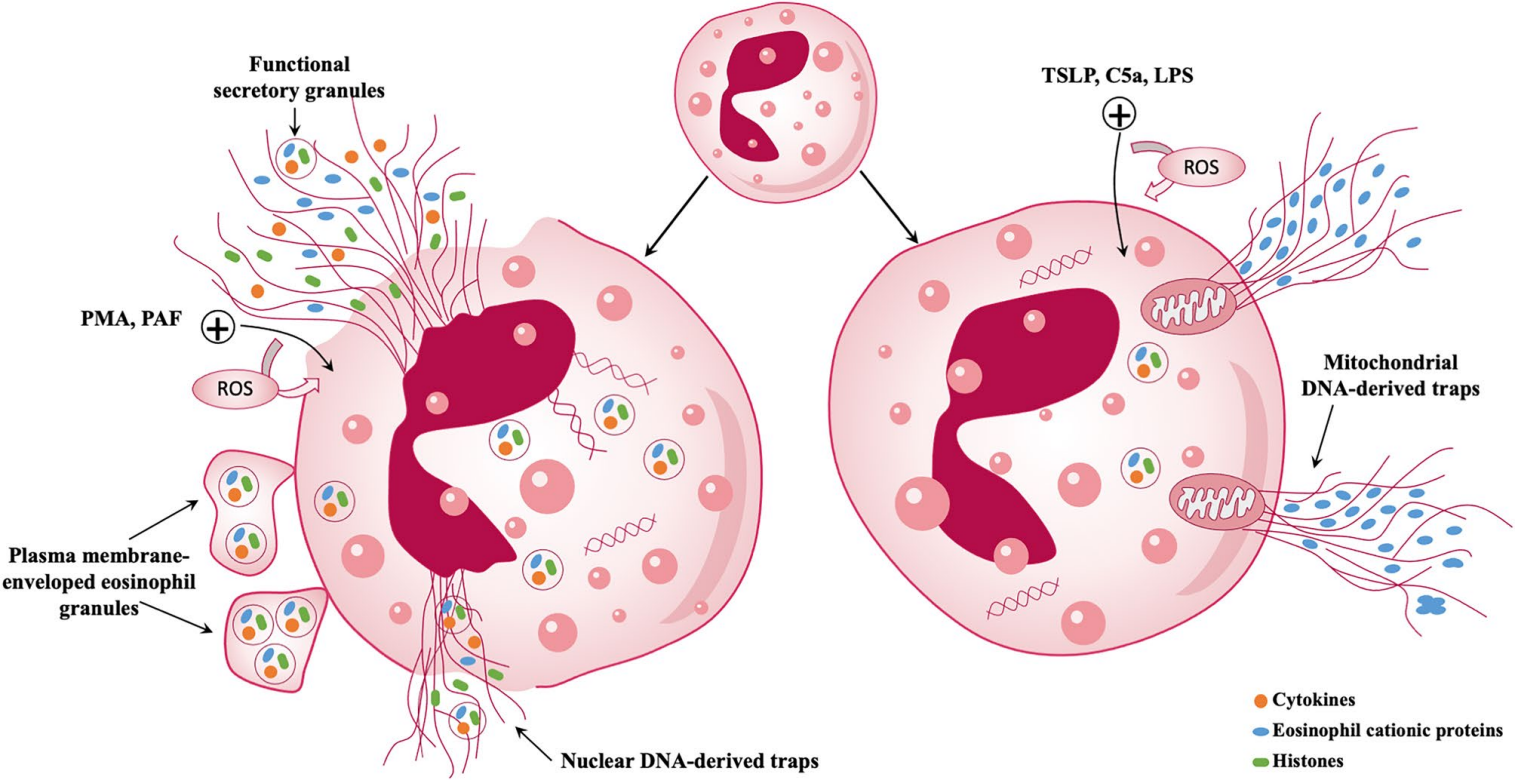
Overview of EETs formation mechanisms in eosinophils: Eosinophils release nuclear DNA associated with ETosis (left) or mitochondrial DNA (right). During ETosis, several eosinophil granules are extruded extracellularly as plasma membrane-enveloped structures, followed by nuclear disintegration, resulting in the formation of intracellular DNA nets. Consequently, plasma membrane disruption leads to the release of both nets derived from nuclear DNA and secretory granules from eosinophils. Eosinophils can also rapidly and independently expel mitochondrial DNA. Both processes rely on reactive oxygen species (ROS) and are initiated by specific triggers

The mechanism of EETs release can be classified based on the source of DNA, either from the nucleus or the mitochondria (Fig. 2). EETs production can be induced by various stimuli, including immunoglobulins (IgG and IgA), platelet-activating factor (PAF), A23187, or PMA, where the DNA originates from the nucleus and is bound to histones. During EETosis, eosinophil granulocytes undergo cytolysis, leading to the dissolution of the nuclear membrane and the mixing of DNA with intact particles. Subsequently, membrane disruption results in the extracellular release of chromatin and associated particles. Alternatively, eosinophil granulocytes can form EETs by ejecting mitochondrial DNA that contains specific eosinophil granulocyte proteins, a process that does not cause eosinophil granulocyte death [7, 70]. However, the origin of DNA from mitochondria has been questioned by some scholars, given the small amount of mitochondrial DNA in cells, but many EETs are formed. Therefore, further studies are required to confirm the classification of EETs release based on DNA source.

EETs can be formed through two different mechanisms: an oxidative NADPH oxidase-dependent mechanism and an oxidative-independent mechanism. When eosinophils are primed with IL-5 or IFN-γ and activated with C5a, LPS, or eotaxin, the production of mitochondrial EETs requires NADPH oxidase-dependent processes. The generation of ROS by NADPH oxidase is also necessary for the release of nuclear-derived EETs in response to stimulation with PAF, IgG/IgA immune complexes, PMA, or TSLP [7, 70, 81]. However, the stimulation of EETs production by lysophosphatidylserine (LysoPS) or *Aspergillus fumigatus* occurs through a ROS-independent mechanism [82, 83]. Human eosinophils also produce the enzyme PAD4, similar to neutrophils, and PAD4-mediated histone citrullination is essential for the formation of EETs induced by LysoPS [82, 84]. However, the role of PAD4-mediated histone citrullination in the formation of EETs triggered by other inducers, such as PMA, PAF, immune complexes, or monosodium urate crystals, is not clear [70, 85].

Recent evidence suggests that EETs may have an importance in host defense. Eosinophils are primarily involved in infections caused by helminthic parasites, but EETs in these infections is not yet well defined. Although previous veterinary research showed that eosinophils release EETs to trap *H. contorta larva*, a known toxic nematode in ruminant animals, the relevance of EETs in this work is unknown due to the low purity of eosinophils extracted [86]. The evidence for the formation of EETs in fungal infections is limited and inconclusive. In a recent clinical case, evidence of EETs forming in a patient diagnosed with allergic bronchopulmonary aspergillosis was reported. Scanning electron microscope images showed that EETs captured *Candida albicans* in vitro, while Muniz et al. demonstrated that *Aspergillus fumigatus* can elicit EETs from eosinophils in vitro in a ROS-independent manner via CD11b binding and activation of the Syk tyrosine kinase pathway [83]. However, the EETs were not found to have fungicidal activity. Eosinophils have also been shown to form EETs in response to bacterial stimulation and can kill bacteria through a phagocytosis-independent mechanism. Activated eosinophils primed with IL-5 and/or IFN-γ have been shown to cast EETs in response to *E. coli* or *S. aureus.* EETs release by lytic eosinophils in response to *S. aureus* has also been shown to be mediated by bacterial virulence factors [7, 9]. Overall, while the exact role of EETs and ETosis in host defense against pathogens is not yet fully understood, the available evidence suggests that they play a significant role in the defense against bacterial or fungal infections and may provide new insights into the development of treatments for infectious diseases.

Despite the demonstrated and crucial role that EETs and EETosis play in host immune response, they have also been linked to a range of eosinophil-related allergy disorders, including CRSwNP, eosinophilic esophagitis, allergic asthma, eosinophilic otitis, and COPD [12, 87–90]. The presence of EETs has been identified in all samples of eosinophilic esophagitis and was found to correlate with the number of infiltrating eosinophils [87]. In asthmatic bronchial biopsies, the amount of EETs containing DNA and colocalizing with MBP was proportional to the number of infiltrating eosinophils [91]. Furthermore, the accumulation of EET-related debris can activate and trigger NETosis, which has been widely observed in individuals with severe exacerbation of COPD [89]. Besides, EETs have also been identified as proinflammatory agents in non-allergic conditions, including the formation of atherosclerotic plaques, thrombosis, Crohn’s disease, and bullous pemphigoid. Platelets-induced EETs enhance thrombus stability through MBP [92]. The potential for EETs to cause tissue damage was observed in bullous pemphigoid, an autoimmune skin disease that results in subepidermal blister formation [93].

## Difference Between NETs and EETs

Although both NETs and EETs are generated via NADPH-oxidase dependent processes and involve nuclear and plasma membrane rupture, these cell types exhibit distinct differences in their granule structures and ETs. NETosis, the granule membranes disintegrate and NE and MPO translocate to the nucleus, where they bind to chromatin and cause nuclear decondensation. This leads to the formation of DNA traps filled with antibacterial granule proteins prior to neutrophil rupture [2, 27]. In contrast, eosinophil DNA traps are associated with intact granules rather than free granule-derived proteins as seen in neutrophil DNA traps. The release of intact granules is a characteristic feature of EETosis [70]. Unlike NETosis, the mixing of nuclear chromatin with granules contents is prevented by rapid plasma membrane disintegration and limited degranulation, resulting in both intact granules and free granule protein DNA traps. The presence of cell-free intact eosinophil granules in DNA traps suggests that EETosis is not just a simple deposition of granule proteins leading to persistent inflammation, but may also have immunoregulatory functions [94]. Remarkably, the surface of the phospholipid bilayer membrane of eosinophil granules expresses cytokine, chemokine, and eicosanoid receptors with ligand-binding competence. Stimulation of granules activates intragranular signaling mechanisms that result in secretion of granule-derived proteins, such as IL-4, IL-6, ECP, and EPO [95, 96]. Certain ETosis-derived free granules produce ECP in response to eotaxin. Consequently, the release of free eosinophil granules into tissue by EETosis is more than just granule protein deposition that causes prolonged inflammation; it could also have immunoregulatory effects.

The overproduction of NETs or EETs lead to an increase in the viscosity of secretions. The DNA traps produced by eosinophils or neutrophils have distinct characteristics, with EETs being composed of more stable and condensed chromatin, while neutrophil DNA traps are composed of stacked nucleosomes and globular domains [70, 88]. The core histones, the most abundant proteins in ETs, help pack two meters of DNA into a small nucleus and serve as targets for enzymes [97]. NETosis releases proteases, including NE, which degrade histones and promote chromatin relaxation, making the DNA more susceptible to nuclease degradation [88, 98]. Proteases appear to play a significant role in the construction and stability of NETs, as neutrophils have higher protease activity compared to eosinophils. As a result, EETs may persist for longer periods of time as they evade proteolytic clearance [88].

## Eosinophil Extracellular Traps in CRS

ECRS is characterized by persistent inflammation of the nasal sinuses with an abundance of eosinophils. This subtype of chronic rhinosinusitis is associated with a higher likelihood of treatment failure with pharmacological interventions, a greater need for surgical intervention, and a higher prevalence of coexisting asthma, in comparison to the Th1/Th17-associated “neutrophilic” form of the disease. Hematopoietic growth factors IL-3, IL-5, and GM-CSF play a crucial role in the growth, differentiation, and maturation of eosinophils [99]. Elevated levels of total serum IgE, IL-4, IL-5, and IL-13 are indicative of tissue eosinophilia in CRS patients. Th2 cytokines contribute to eosinophilia by influencing the differentiation, survival, and activation of eosinophils. Research has shown that eosinophils from nasal polyps exhibit elevated levels of CD69 mRNA, a marker of cellular activation, compared to peripheral blood eosinophils, indicating that the eosinophils in nasal polyps are activated [100, 101]. Activated eosinophils release granule proteins, including MBP, EPX, ECP, and EDN, which can cause tissue damage and remodeling in the nasal mucosa. Eosinophil-derived CLCs are comprised of galectin-10, a protein found in high abundance in the cytoplasm of eosinophils, which may serve as a biomarker of eosinophilic and type 2 inflammation [102, 103]. Recent studies have used CLCs mRNA and protein levels in nasal secretions or tissues as predictive indicators for recurrent CRS with nasal polyps and sensitivity to glucocorticoids [90, 103–105]. In conclusion, the activation and accumulation of eosinophils, along with the release of their granule proteins, have been found to contribute negatively to the development and progression of ECRS.

The formation of EETs has been observed in the tissues of both CRSwNP and CRSsNP patients with type 2 inflammation. Research has shown that patients with CRSwNP have higher levels of IL-5, eotaxin, IL-33, and TSLP, as well as persistent colonization with Staphylococcus aureus, which are all potential triggers for EETs formation. EETs were found in inflamed nasal mucosa with eosinophil infiltration in both ECRS and non-ECRS patients. These findings may be related to the histological heterogeneity of CRS [9, 81]. The production of EETs is strongly correlated with the severity of chronic rhinosinusitis, regardless of the presence of nasal polyps [12]. In CRSwNP, EETs are predominantly found in subepithelial regions with epithelial barrier defects, which can lead to the entrapment of *S. aureus* [9, 81]. EETs are also highly present in mucus from patients with eosinophilic CRS, increasing the viscosity of the mucus [88]. However, the exact mechanism underlying the formation of EETs in CRS is still not fully understood.

Despite the fact that EETs and associated granule proteins have protective functions for the host, they can also provoke epithelial barrier dysfunction and airway remodeling [9, 88]. In active eosinophilic esophagitis, Simon et al. observed that eosinophilic infiltration and the release of EETs in the esophagus can lead to epithelial barrier abnormalities, increased production of antimicrobial peptides, and epithelial-derived cytokines [75]. Eosinophils are believed to be recruited to regions of epithelial disruption to generate EETs and protect against infections [9]. However, the direct impact of EETs on nasal epithelial damage has not been established. In addition, EETs have been found to significantly increase the release of IL-6 from human primary small airway epithelial cells [106]. IL-6 affects ciliary beating in human nasal epithelium ciliated cells, which are involved in the growth of nasal polyps (NP) [107]. MBP, which is released by eosinophils, can also induce airway remodeling by increasing the expression of epithelial transforming growth factor beta (TGF-b) and matrix metalloproteinase 1 (MMP-1), as well as causing subepithelial fibrosis [108]. The release of ECP, a hallmark of eosinophilic inflammation, may also contribute to TGF-b-mediated fibrosis in CRS [109]. In a recent study, EDN stimulation of human nasal epithelial cells resulted in an increase in MMP-9 expression as determined by RNA sequencing [110–112]. MMP-9 levels are higher in nasal polyps and are believed to contribute to tissue remodeling [113]. In conclusion, EETs and associated granule proteins may play a critical role in the development of persistent eosinophilic inflammation in CRS through epithelial barrier dysfunction and tissue remodeling.

There is increasing evidence that EETs have a function in CRS and contribute to Th2 inflammation.The synthesis of EETs underlies the deposition of CLCs, the crystallized form of galectin-10 [8, 90]. For the first time, Ueki et al. demonstrated CLCs formation is tightly associated with EETs cell death. In clinical circumstances, the presence of CLCs or a rise in the local galectin-10 concentration might serve as an alternative sign of extensive occurrences of EETosis [8, 114]. EETs and CLCs are abundant in the mucosa and mucus of both CRSsNP and CRSwNP and are linked with type 2 inflammation [67, 90, 115]. A study indicated that the mRNA expression of CLCs was a better predictor of eosinophilic CRSwNP than the ratio of blood eosinophils, suggesting that CLCs mRNA could serve as a potential biomarker for diagnosing and classifying endotypes of CRS [116]. CLCs protein in nasal secretions may serve as a promising noninvasive biomarker to predict CRSwNP recurrence [104]. In addition, Choi et al. revealed that higher levels of peripheral EETs-forming eosinophils and ILC2s were observed in severe asthmatics compared to non-severe asthmatics, with a positive correlation to higher lung IL-33 and TSLP levels. An in vivo experiment demonstrated that EETs can activate ILC2s in lung tissues by stimulating the airway epithelium to produce IL-33 and TSLP, which can be inhibited by anti-IL-33 antibody treatment [117]. Mast cells were reported to release histamine in response to stimulation by MBP and ECP [118]. EDN can also activate the TLR2-MyD88 signaling pathway in dendritic cells, leading to sustained Th2 immune responses [119].

The presence of eosinophilic and neutrophilic inflammation in CRS can no longer be considered separate processes. Patients with a mixed granulocytic phenotype in CRS, who exhibit both eosinophilic and neutrophilic inflammation, have been found to have more severe tissue inflammation and a higher overall inflammatory burden than those with predominantly eosinophilic or neutrophilic CRS. This is indicated by worse CT scores, reduced olfactory function, decreased disease-specific quality of life, and a higher symptom burden [120, 121]. The development of neutrophil infiltration has been linked to the formation of EETs and CLCs, hallmarks of eosinophilic inflammation, in severe type 2 CRS patients. Furthermore, research has shown that stimulation of CLCs in vitro can lead to increased neutrophil recruitment towards epithelial cells [57, 67]. CLCs contribute to inflammation by activating the NLRP3 inflammasome in macrophages, resulting in IL-1β-driven inflammation, whereas soluble galectin-10 has anti-inflammatory effects [67, 122]. However, only CLCs induce those pro-inflammatory effects in CRSwNP, whereas soluble galectin-10 exhibited anti-inflammatory effects [67]. The use of CLC-dissolving antibodies has been found to suppress airway inflammation, goblet-cell metaplasia, bronchial hyperreactivity, and IgE synthesis in a humanized mouse model induced by CLC or house dust mite inhalation [90]. Additionally, eosinophil-derived MBP has been shown to activate neutrophils [123]. These findings suggest that the interaction between eosinophils and neutrophils may play a critical role in maintaining the mixed inflammation observed in individuals with severe type 2 CRS through the formation of EETs and CLCs.

## Treatment

Inflammation associated with ETs can be mitigated by inhibiting the formation of ETs, blocking the proteins that decorate ETs, or removing ETs that have already been released. Research is ongoing into the use of inhibitors that can block NETs formation and compounds that can break down NETs as a potential treatment for inflammatory illnesses [48]. Vargas et al. discovered that glucocorticoids effectively reduced NETs formation both in vitro and in vivo in the lungs of asthmatic horses [124]. Asthma patients who were receiving inhaled glucocorticoid (ICS) medication displayed lower circulating NETs levels compared to those who did not use ICS or used it only occasionally [125]. However, some prior trials have indicated that corticosteroids may not suppress NETs-mediated airway inflammation [66, 91, 126]. The amount of eDNA in neutrophils treated with different doses of dexamethasone was similar to that in untreated control neutrophils. Further investigation into new biologics to reduce ETs in upper airway inflammation is essential. The use of recombinant human protease inhibitors, such as NE inhibitors, and DNase to neutralize and break down NETs-derived DNA and mediators can effectively lower their proinflammatory effects [127]. Inhibiting NE has been shown to prevent NETs-induced disruption of the integrity between endothelial cells [128]. Moreover, blocking NE has been shown to reduce rhinovirus-induced airway hyperreactivity in a mouse model of asthma [79]. DNase, which disintegrates the chromatin in NETs, has shown potential as a method for inhibiting NET formation and activity [129]. Additionally, the use of anti-histone antibodies has proven effective in treating autoimmune disorders [130]. PAD4 has been identified as a potential target for reducing NET-mediated inflammation in various mouse models. Cl-amidine, an inhibitor of PAD4, has also been shown to inhibit histone citrullination, a key step in NETosis [130]. It is important to note that NETosis may not always be dependent on PAD4, and the effectiveness of PAD4 inhibitors may vary across species. Other compounds with the potential to prevent NETosis are being researched. The inhibition of NADPH oxidase has been shown to prevent suicidal NETosis in vitro, but research on experimental murine models of SLE and gout, which lacked NADPH oxidase, revealed more severe disease [50, 131]. It is crucial to continue researching and understanding the regulation and balance of NETs formation, inhibition, and degradation via the use of NETs inhibitors in order to prevent harm to the patient's immune system.

Currently, there is limited research into the treatment of EETs, with a focus on preventing EETs production. To date, anti-IL-5 or IL-5R antibodies have been approved as adjunctive treatment for severe ECRS patients, where eosinophil activation and EETs formation may be elevated due to high levels of IL-5 and other factors [106, 132]. These biologics have the potential to effectively prevent type 2 inflammation in CRS patients by blocking eosinophil activation [7]. Anti-TSLP antibodies have also been shown to reduce asthma exacerbations and improve lung function in patients with uncontrolled severe asthma [133]. The role of TSLP in stimulating EETs production, further investigation is needed to determine whether anti-TSLP antibodies can decrease EETs-induced inflammation in CRS [81]. Antibodies targeting the crystallization interface of galectin-10 have been demonstrated to effectively reduce illness in a humanized mouse model of asthma [90]. These antibodies may be a candidate biologic for CRS, but it remains to be determined through additional clinical trials if they can effectively inhibit EETs production.

## Conclusion

CRS is a complex condition with a diverse pattern of inflammation, characterized by elevated levels of cytokines, chemokines, and lipid mediators, as well as the infiltration of inflammatory cells. Although NETs and EETs have protective functions for the host, they can also exacerbate eosinophilic or neutrophilic inflammation in CRS by damaging surrounding tissues and disrupting the epithelial barrier. The exact mechanisms of NETs and EETs formation and their roles in CRS are not fully understood, and further research is needed to explore the presence of mixed eosinophilic-neutrophilic inflammation in CRS. Currently, ETs are known to occur in neutrophils and eosinophils, as well as in other innate immune cells such as macrophages, basophils, and mast cells. However, the role of ETs in other cell types in CRS has yet to be investigated. The inhibition of NETs and EETs formation may represent a potential target for treating CRS.

## Data Availability

This is a comprehensive literature review that solely relies on publicly accessible publications, rather than primary data. Access to these materials is dependent on the reader's institutional licensing.
